# Effectiveness of Community-Based Interventions Programs in Childhood Obesity Prevention in a Spanish Population According to Different Socioeconomic School Settings

**DOI:** 10.3390/nu12092680

**Published:** 2020-09-02

**Authors:** Ana M. Puga, Alejandra Carretero-Krug, Ana M. Montero-Bravo, Gregorio Varela-Moreiras, Teresa Partearroyo

**Affiliations:** Departamento de Ciencias Farmacéuticas y de la Salud, Facultad de Farmacia, Universidad San Pablo-CEU, CEU Universities, Urbanización Montepríncipe, Alcorcón, 28925 Madrid, Spain; anamaria.pugagimenezazca@ceu.es (A.M.P.); alejandra.carreterokrug@ceu.es (A.C.-K.); amontero.fcex@ceu.es (A.M.M.-B.); gvarela@ceu.es (G.V.-M.)

**Keywords:** weight management, obesity, overweight, body composition, children, socioeconomic status

## Abstract

Overweight and obesity amongst childhood are currently global health issues. However, this is the best stage of life to prevent diseases and to promote healthy habits. In our study, we evaluate the effectiveness of the THAO Salud Infantil, a community-based intervention program, by means of a cross-sectional study carried out from 2009 to 2019 surveying children aged 3 to 12 years old (*n* = 27,686). During the study timeframe, overweight and obesity prevalence, according to both the International Obesity Task Force and Orbegozo Foundation criteria, showed a downward trend. Differences in the anthropometric variables were observed from the beginning to the end of the study, mainly in girls. Analysis of the influence of the socioeconomic status revealed that children from families with lower incomes are in greater risk of suffering from overweight and obesity and showed lower effectiveness of the actions proposed by the program. The overall results of the study confirmed the effectiveness of community-based interventions in terms of childhood overweight/obesity prevention.

## 1. Introduction

Overweight and obesity in childhood and adolescence have recently became global health issues [1]. According to the World Health Organization (WHO), obesity is currently the fifth leading global risk factor for mortality [2]. Moreover, the WHO reported that in 2016, more than 1.9 billion adults and over 340 million children and adolescents aged 5–19 years worldwide presented overweight. Of particular concern is the fact that overweight and obesity prevalence in children and adolescents has risen dramatically from just 4% in 1975 to just over 18% in 2016 [3]. Regarding Spanish data, the latter National Health Survey revealed that 28.5% of children aged 2–17 years were overweight or obese [4]. Furthermore, data from the “Anthropometric data, macronutrients and micronutrients intake, practice of physical activity, socioeconomic data and lifestyles in Spain” (ANIBES) study revealed that in the population aged 9 to 17 years, the prevalence of overweight and obesity was 27.9% and 8.8%, respectively [5]. Likewise, the ENPE (*Estudio Nutricional de la Población Española*) study revealed that overweight and obesity prevalence in Spanish populations aged 3 to 24 years exceeded 30%, whereas 16% were overweight and had concomitant abdominal obesity [6]. This worrying situation has led to the appearance in children and adolescents of pathologies previously observed only in adults, such as diabetes mellitus, hypertension, coronary heart disease and/or fatty liver [7,8,9], which result in not only adulthood mortality and premature death [10] but also a worse quality of life [11]. Moreover, childhood obesity is associated with somatic health problems as well as negative self-evaluation, bullying, social stigma, depression, and anxiety, resulting in a negative impact on physical and psychosocial health in children and adolescents [11].

Overweight and obesity are the result of an imbalance between energy intake and expenditure that leads to positive energy balance being associated with lifestyle (activity level) and dietary intake [12]. However, currently it is well known that obesity is influenced by other factors such as genetic background, composition of gut microflora, sedentary behavior, endocrine diseases, hydration status and/or socioeconomic position, among others [1,13,14,15]. Currently, there is great evidence available in the literature suggesting the association between socioeconomic status and the development of childhood obesity. Furthermore, the widening gap in obesity rates between the low and high socioeconomic status groups is becoming increasingly apparent [16,17]. This could be associated with the financial difficulties related to the lower socioeconomic status, which make healthy lifestyle choices less accessible, indirectly promoting unhealthy lifestyle choices such as eating unhealthy and generally high density food, lack of physical activity and fewer opportunities for healthy growth and development [18]. Moreover, the present and future Covid-19 pandemic consequences may lead to an even more dramatic situation since many families may become highly economically vulnerable.

Interestingly, schools play a key role in obesity prevention, owing to their long-term and in-depth contact with children. In fact, school environment, policies, curriculum, extra-curricular activities, and personnel may positively influence children’s lifestyle behaviors [19]. In addition, the traditional role of families in nutrition education is becoming more complex due to the lack of time, work constraints, etc. Therefore, the majority of interventions focused on childhood obesity prevention are school-based [20]. One example is the WHO’s Health Promoting Schools framework, focused on creating school environments conducive to health and healthy behaviors [21]. On the other hand, community-based interventions programs focused on the prevention of childhood obesity acting at different key sectors of childhood development, namely family, health professionals, sports, media, shops, market and, again, schools. However, there is scarce knowledge of the effectiveness of these programs, mostly due to their inconstancy and insufficient follow-up. For example, results from the *Fleurbaix Laventie Ville Santé* study carried out in France revealed that the implemented community-based intervention program led to less weight gain in the intervention towns than in the control town [22]. However, results of Gomez et al. [23] showed that these kind of programs do not improve weight development, diet quality and physical activity in the short term. Therefore, the aim of our study was to evaluate the effect of a community-based intervention program on the anthropometric measurements and indices in a large and standardized cohort of Spanish children, and to analyze the relative contribution of the school type, as a marker of the socioeconomic status, in overweight and obesity prevalence and trends.

## 2. Materials and Methods

The *THAO Salud Infantil* Program is a community-based intervention program based on the *Fleurbaix Laventie Ville Santé* study (FLVS study) and carried out within the EPODE Program (*Ensemble Prévenons l’Obesité Des Enfants*) International Network (France). It is focused on the prevention of childhood obesity by means of the promotion of healthy life habits. It was developed by the THAO Foundation through the municipalities in order to promote healthy life habits in children aged 3 to 12 years [24]. The THAO program was mainly devoted to longitudinally evaluate anthropometric measurements and the efficacy of different actions organized to prevent obesity. Only five towns were initially selected in Spain as pilots for global evaluation and targeted actions. Villanueva de la Cañada (Madrid, Spain) was therefore selected as a pilot city, where the *THAO Salud Infantil* Program was implemented in 2007. However, the initially planned *THAO Salud Infantil* Program ended in 2016, even though Villanueva de la Cañada council continued it under the name of “*Programa de prevención del sobrepeso y la obesidad-Estrategia NAOS en Villanueva de la Cañada*”(Program for the prevention of overweight and obesity-NAOS strategy in Villanueva de la Cañada). Program implementation was led by the city council and included different intervention activities, such as monitoring of menus in school canteens, organization of healthy breakfasts and breaks, development of school gardens and implementation of other activities related to the heavy promotion of physical activity and the consumption of fruits and vegetables, among others.

This school-based cross-sectional study was carried out during academic years 2009–10, 2010–11, 2011–12, 2012–13, 2013–14, 2014–15, 2015–16, 2016–17, 2017–18 and 2018–19, surveying schoolchildren aged 3 to 12 years old of both genders (*n* = 27,686) from state, charter and private schools in Villanueva de la Cañada (Madrid, Spain). Specifically, volunteers were selected among the students of the indicated ages. The ethical approval was granted by the Clinical Research Ethics Committee of the CEU San Pablo University (Madrid) (ethical code 121/16/07). Moreover, the study was performed in accordance with the ethical standards laid down in the 1964 Declaration of Helsinki and its later amendments. Participants were informed about the goals of the study, and parental written consent on behalf of each participant was obtained prior to his or her inclusion in the study.

### 2.1. Study Protocol

The study involved an anthropometric evaluation of the subjects that included the measurement of weight, height, and waist circumference. Weight was measured by means of an electronic scale (Seca 710 scale, Seca gmbh and Co, Hamburg, Germany) with an accuracy of 100 g, and height was measured to the nearest 0.5 cm using a stadiometer (Seca 213 Telescopic Height Rod for Column Scales, Seca gmbh, Hamburg and Co, Hamburg, Germany). Finally, waist circumference (WC) was measured with a flexible steel tape (CESCORF, Porto Alegre, Brazil). All anthropometric measurements were made according to the recommendations of the International Standards for Anthropometric Assessment (ISAK) [25] by level I and II accredited anthropometrists. Body mass index (BMI) was calculated as the relation between weight (kg) and the square of the height (m) [26] and compared to the international cut-offs established by the International Obesity Task Force (IOTF) [27] and the Spanish cut-offs established by the Faustino Orbegozo Foundation (OF) [28], to define underweight, normal weight, overweight and obesity. BMI z-score was calculated by expressing a child’s BMI relative to children in the OF growth charts [28].

### 2.2. Statistical Analysis

Results are presented as median (interquartile range) per group or as percentage. Variables were tested for normality using a Kolmogorov–Smirnov test. Non-parametric data were analyzed by the Kolmogorov–Smirnov test. Chi square test was used to compare the overweigh and obesity prevalence between years and type of school. Differences were considered significant at *p* < 0.05. All statistical analyses were performed using SPSS 24.0 Software (IBM Corp., Armonk, NY, USA).

## 3. Results

The study population included 27,686 children (13,925 boys and 13,761 girls). The distribution of the study population in each academic year is shown in Table 1.

Table 2 and Table 3 summarize the anthropometric characteristics of boys and girls at baseline and the end of the nine–years follow up, respectively. In boys, WC, weight, height, and BMI values were, on average, around 50–70 cm, 15–43 kg, 97–149 cm and 16–19 kg/m^2^, respectively. No statistically significant differences were found in boys between anthropometric data at baseline and after the follow up, except for those aged 6, 7 and 12 years old (Table 2), where statistically significant differences for WC, BMI and BMI z-score (*p* ≤ 0.01 in boys aged 6 and *p* ≤ 0.05 in those aged 7 and 12) were determined between baseline and after 9-years follow up. Moreover, in boys aged 7 years, statistically significant differences were found for weight (*p* ≤ 0.05) at baseline and at the end of the study, whereas in those aged 12 years, significant differences were observed in WC at baseline and 9 years follow up (*p* ≤ 0.05). On the other hand, in girls (Table 3), WC, weight, height and BMI values were, on average, around 49–69 cm, 14–44 kg, 95–152 cm and 15–19 kg/m^2^, respectively, and these values were similar after 9 years follow up. Of interest, in girls aged 4, 6, 9, 10, 11 and 12-years, WC was significantly higher in 2010 than in 2019 (*p* ≤ 0.05). In addition, in girls aged 4 and 7 years, statistically significant differences in BMI and BMI z-score (*p* ≤ 0.001 and *p* ≤ 0.05, respectively) were observed from the beginning to the end to the study.

**Figure 1:**
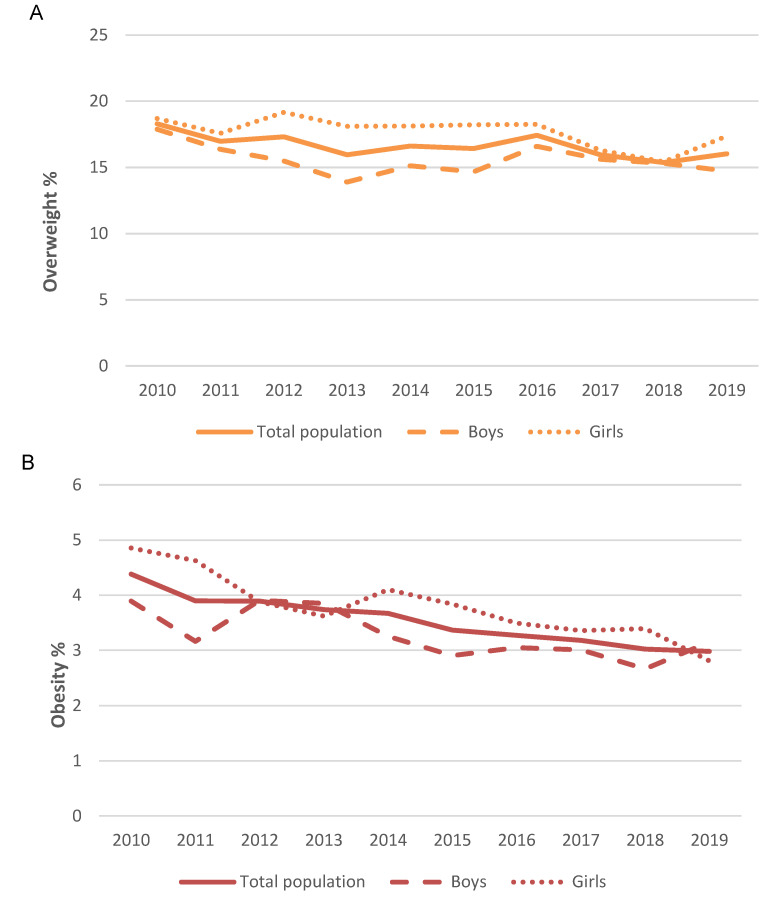
Prevalence of overweight (**A**) and obesity (**B**) among Spanish children by International Obesity Task Force (IOTF) criteria in the 2010–2019 timeframe.

**Figure 2:**
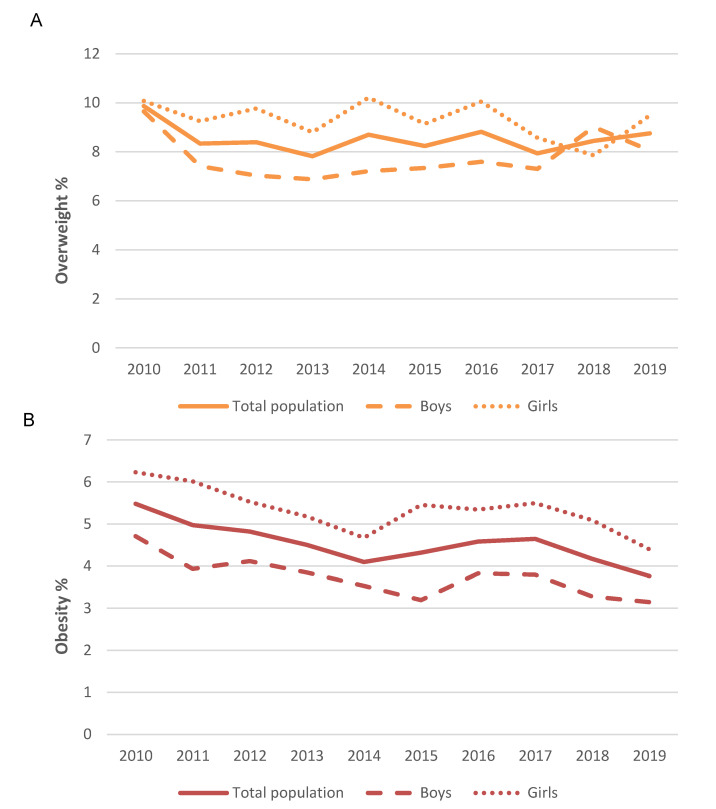
Prevalence of overweight (**A**) and obesity (**B**) among Spanish children by Faustino Orbegozo Foundation (OF)criteria in the 2010–2019 timeframe.

Across the study timeframe, the prevalence of overweight according to the IOTF reference values (Figure 1) tended to decrease from a peak of 18.3% (2010) to the lowest value in 2018 (15.4%) (Figure 1A). According to the Spanish OF criteria, a similar decreasing tendency in overweight prevalence was observed from 2010 (9.9%) to 2019 (8.9%) (Figure 2A). Regarding obesity prevalence, it followed the same trend with a decrease in obesity prevalence that ranged from 4.4% (2010) to 3.0% (2019) according to IOTF criteria (Figure 1B) and from 5.5% in 2010 to 3.8% in 2019 according to OF criteria (Figure 2B). This trend was also observed when the total population was stratified by sex (Figure 1 and Figure 2).

**Figure 3:**
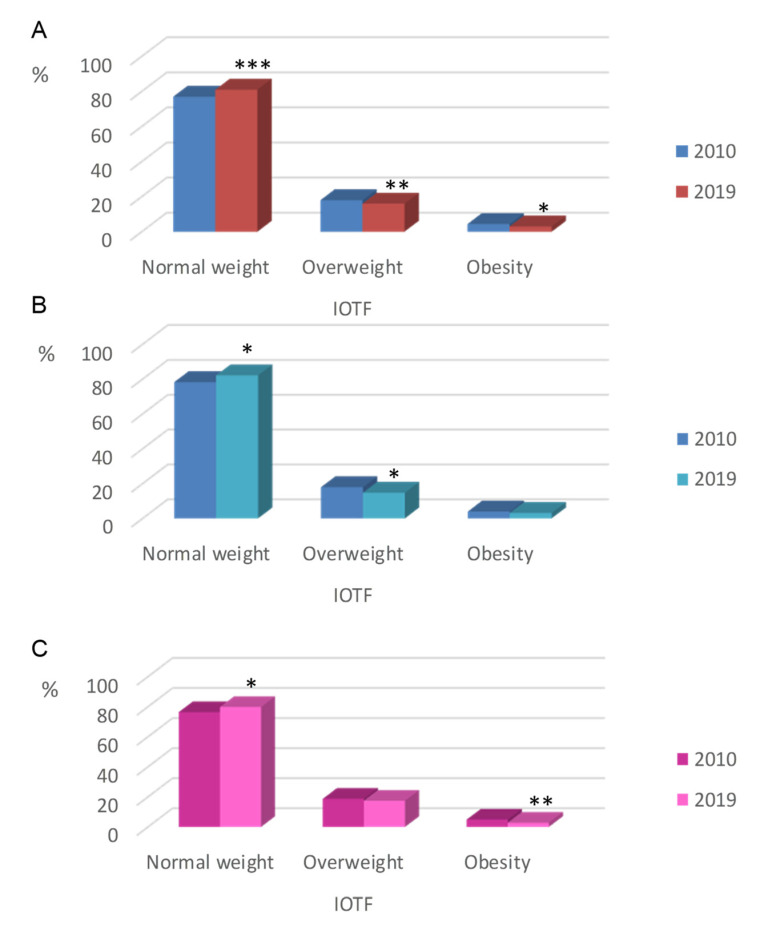
Prevalence of normal weight, overweight and obesity in Spanish children by International Obesity Task Force (IOTF) reference. (**A**) Total population. (**B**) Boys. (**C**) Girls. * *p* ≤ 0.05 vs. year 2010 (Chi-Square test); ** *p* ≤ 0.01 vs. year 2010 (Chi-Square test) *** *p* ≤ 0.001 vs. year 2010 (Chi-Square test).

Percentages of normal weight, overweight and obesity in the study population at baseline and at the end of the study were presented by IOTF reference values (Figure 3). From 2010 to 2019, the prevalence of normal weight among children aged 3–12 years varied from 77.3% to 81.0%, whereas the prevalence of overweight (including obesity) ranged from 22.7% to 19.0%. When compared to baseline, according to IOTF, the global age standardized prevalence of normal weight in children increased up to 3.7% (*p* ≤ 0.001), whereas the overweight and obesity prevalence in children decreased by 2.3% (*p* ≤ 0.05) and 1.4% (*p* ≤ 0.01), respectively (Figure 3A). Data from 2019 showed that the prevalence of overweight (including obesity) tended to decrease in both sexes with respect to 2010 data; this trend was even more remarkable in boys (from 21.8% in 2010 to 17.8% in 2019) (Figure 3B) than in girls (from 23.6% in 2010 to 20.2% in 2019) (Figure 3C).

**Figure 4:**
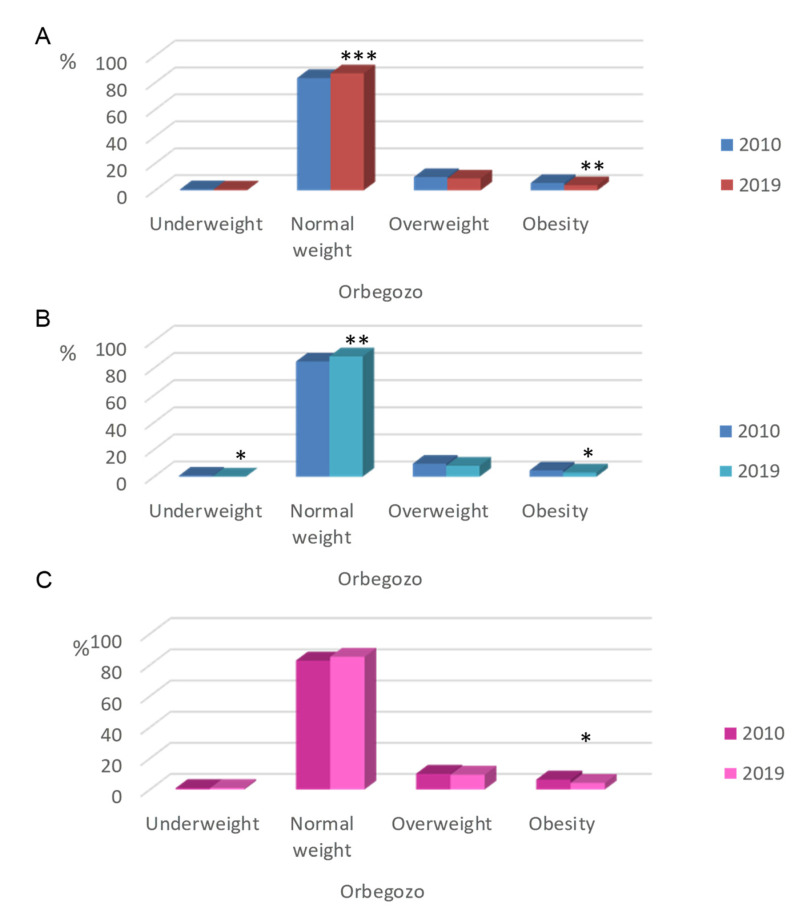
Prevalence of underweight, normal weight, overweight and obesity in Spanish children by Faustino Orbegozo Foundation (OF) reference values. (**A**) Total population. (**B**) Boys. (**C**) Girls. * *p* ≤ 0.05 vs. year 2010 (Chi-Square test); ** *p* ≤ 0.01 vs. year 2010 (Chi-Square test); *** *p* ≤ 0.001 vs. year 2010 (Chi-Square test).

From children studied in 2010, according to OF criteria (Figure 4), 15.4% were overweight or obese, of which 9.9% were overweight (9.6% of boys, 10.1% of girls) and 5.5% were obese (4.7% of boys, 6.3% of girls). Regarding data from 2019, both overweight and obesity prevalence was lower than in 2010 (8.8% and 3.8%, respectively), with the obesity prevalence higher in girls (4.2%) than in boys (3.1%). Conversely, from 2010 to 2019, normal weight prevalence significantly increased (*p* ≤ 0.01) from 83.4% to 86.9%. In the total population, the prevalence of underweight slightly decreased from 0.8% in 2010 to 0.6% in 2019 (from 0.8% in 2010 to 0.2% in 2019 in boys, whereas in girls no change was observed). Of interest, this change was lower than the rise found in obesity.

Table 4 shows the global prevalence of underweight, normal weight, overweight and obesity from Spanish children according to IOTF and OF criteria based on the school type. Both overweight and obesity prevalence were significantly higher in state schools followed by charter schools and private schools (*p* < 0.01) according to both criteria. However, no significant differences in underweight prevalence were determined based on OF criteria.

Finally, the proportions of underweight, normal weight, overweight and obese children by school and stratified by sex are shown in Table 5. No significant differences were observed in the proportion of boys that presented normal weight and overweight according to IOTF criteria, between charter and private schools. However, these types of schools presented lower prevalence of overweight than state schools and, therefore, higher prevalence of normal weight (*p* < 0.01). Regarding obesity, state schools showed significantly higher prevalence than charter schools and these, in turn, significantly higher obesity prevalence than private schools. However, in girls, according to the IOTF criteria, no significant differences between state and charters schools were found in terms of overweight prevalence, but when compared to full private schools, this was significantly lower. Nevertheless, obesity prevalence in girls based on the same criteria was significantly higher in state schools followed by charter and private schools, the ones with the lowest obesity rates. Data analysis according to OF criteria revealed that in boys, as observed before, normal weight prevalence in charter and private schools were significantly higher than that of state schools, whereas both overweight and obesity prevalence was higher in state schools than in private ones (*p* < 0.01). On the other hand, in girls by OF criteria, normal weight prevalence was significantly higher than that of charter schools and this, in turn, was significantly higher than that of state schools. The inverse trend was observed in terms of obesity prevalence, with the highest obesity prevalence observed in the public schools.

## 4. Discussion

Overweight and obesity have a multifactorial etiology involving genetic predisposition and multiple environmental factors, which include energy intake, activity level, composition of gut microflora, sedentary behavior, endocrine diseases, hydration status and/or socioeconomic position, among others [1,13,14,15]. The first and key step to develop prevention and repair strategies for intervention before damage becomes irreversible is to develop programs to prevent childhood obesity. Due to the multifactorial causes of obesity, community-based intervention programs, such as the THAO [24] and other related programs, seem to be a promising alternative. However, to date, the available evidence of the effectiveness of this kind of complex intervention is moderate [29]. During the study timeframe, analysis of the anthropometric data of children and adolescents revealed a trend of improvement of anthropometric variables, especially in girls. Thus, a decrease in both BMI and WC, indicative of abdominal fat distribution, were observed. These findings are different than those from Gomez et al. [23] who reported that two academic years’ worth of a community-based intervention program reported no significant differences in terms of weight development, obesity incidence or changes in diet quality. These differences could be attributed to the need for a longer follow-up period to observe improvements in terms of body composition of the childhood. In fact, it has been stated that long follow-up periods (from at least 24 to 48 months) accompanied by actions at multiple settings, including school, family and the community are needed for the success of community-based interventions programs [29].

Nowadays, owing to the absence of a widely-shared consensus, it is highly recommended that prevalence studies consider several reference values [30], specifically international standards such as the ones from the IOTF and at a national level the OF criteria. Thus, it is well known that results and their comparison in terms of overweight and obesity prevalence is highly dependent on the methodology used [31,32]. Therefore, in our study both criteria were used to determine overweight/obesity prevalence at the baseline and at the end of the follow-up period. At the beginning of the study, overweight (including obesity) prevalence were 22.7% and 15.4% based on the IOTF and the OF criteria, respectively. The estimated prevalence was lower than those obtained in the ANIBES [5] or ENPE [6] studies, but similar, especially to the one calculated using the IOTF criteria than the reported by the Spanish National Health Survey [4]. However, both in the ENPE and the ANIBES studies as in ours, the higher overweight prevalence was obtained based on the IOTF criteria followed by the OF. Overweight and obesity prevalence in the targeted city was lower than in other areas of Spain, probably due to the adherence of this city to the “Health Promotion and Prevention Strategy” launched by the Spanish National Health System. Likewise, this city was also involved in the main health networks at international, national, and local levels, such as the WHO European Healthy Cities Network, the Spanish Healthy Cities Network and the Municipal Health Network of Madrid. The effectiveness of the program was confirmed by the decrease of overweight and obesity prevalence during the study follow-up, disregarding the criteria used. Hence, overweight prevalence from the baseline to the end of the study decreased by 2.4% and 1.0%, whereas obesity prevalence decreased by 1.4% and 1.7% attending to IOTF and OF criteria, respectively.

On the other hand, it is widely accepted that socioeconomic status plays an important role in overweight/obesity development [33,34]. School type could be used as indicator of the socioeconomic status of the children [35]. The educational system in Spain involves the existence of three types of schools: (i) state/public schools, fully funded by the Government; (ii) charter schools, privately run but partially funded by the Government, where families must pay certain fees and (iii) private schools, fully funded by family fees, which are considerably higher than those for charter schools. Therefore, children belonging to families with medium-high and high socioeconomic status usually study in charter or private schools. On the other hand, state school students belong, generally, to families with low socioeconomic resources. Results of our study revealed statistically significant differences in overweight and obesity prevalence between private (either charter or fully private schools) and state schools. Likewise, private schools showed higher normal weight prevalence than state schools. These findings could be attributed to fact that lifestyle patterns of children from low socioeconomic status are prone to high levels of sedentary behaviors as stated by the systematic review from Leech et al. [36]. Conversely, lifestyle patterns characterized by high physical activity/sports participation were significantly associated with family income [37,38]. Regarding Spanish data, the ANIBES study revealed that healthier lifestyle patterns included lower proportions of children and adolescents from low socioeconomic status backgrounds, whereas low physical activity and poorer diet was observed in families with low socioeconomic status [5].

Furthermore, childhood is the best stage of life to prevent diseases and to promote healthy habits and, thus, the prevention of obesity is of major importance. However, socioeconomic status plays a crucial role that is important to consider. In fact, results of a longitudinal study carried out in Finland for over thirty years reported that higher socioeconomic status children in their adulthood showed healthier lifestyle patterns, including lower meat consumption, higher fish consumption, higher diet score and a higher physical activity index [39]. Therefore, greater attention should be paid to lifestyle behaviors of children of families with lower incomes. In addition health and educational programs aimed at preventing childhood obesity should also focus at other modifiable risk factors such as healthy birth weight, parental smoking and eating meals as a family [40], especially having in mind the higher overweight/obesity prevalence, as stated in our study.

The main strength of our study is the large sample size (*n* = 27.686) as well as the long term follow up of the participants. Moreover, accredited anthropometrists (level I and II. ISAK) performed and collected the anthropometric measurements. However. the study also shows some limitations. For example, a limited amount of biological and behavioral variables were included as potential predictors of obesity development. Hence, we could not explore the influence of factors as maternal obesity, smoking at home, TV time, sleep duration, birth weight, breastfeeding and dietary and physical activity habits.

## 5. Conclusions

In conclusion, this study confirmed the effectiveness of community-based interventions, such as the THAO program at the local level (Villanueva de la Cañada municipality), in terms of childhood overweight/obesity prevention. Likewise, differences observed in prevalence according to children’s socioeconomic status confirmed the importance of health programs, especially in children from low-income families, owing to their great susceptibility to being overweight/obese. The latter may be of critical importance in a time of socioeconomical crisis associated with Covid-19 pandemic consequences.

## Figures and Tables

**Table 1:** Study population distribution from 2010 to 2019.

Year 2010	Total Population (*n*)	2717
	Boys (*n*)	1337
	Girls (*n*)	1380
**Year 2011**	**Total Population (*n*)**	**2593**
	Boys (*n*)	1296
	Girls (*n*)	1297
**Year 2012**	**Total Population (*n*)**	**2801**
	Boys (*n*)	1408
	Girls (*n*)	1393
**Year 2013**	**Total Population (*n*)**	**2864**
	Boys (*n*)	1455
	Girls (*n*)	1409
**Year 2014**	**Total Population (*n*)**	**2806**
	Boys (*n*)	1415
	Girls (*n*)	1391
**Year 2015**	**Total Population (*n*)**	**2733**
	Boys (*n*)	1377
	Girls (*n*)	1356
**Year 2016**	**Total Population (*n*)**	**2813**
	Boys (*n*)	1410
	Girls (*n*)	1403
**Year 2017**	**Total Population (*n*)**	**2797**
	Boys (*n*)	1397
	Girls (*n*)	1400
**Year 2018**	**Total Population (*n*)**	**2878**
	Boys (*n*)	1464
	Girls (*n*)	1414
**Year 2019**	**Total Population (*n*)**	**2684**
	Boys (*n*)	1366
	Girls (*n*)	1318

**Table 2:** Anthropometric variables in boys at baseline and after 9 years.

Baseline							After 9 Years						
Age (year)	*n*	Waist Circumference (cm)	Weight (kg)	Height (cm)	BMI (kg/m^2^)	BMI Z-Score (kg/m^2^)	Age (year)	*n*	Waist Circumference (cm)	Weight (kg)	Height (cm)	BMI (kg/m^2^)	BMI Z-Score (kg/m^2^)
3	52	51.7	15.8	97.4	16.4	0.1	3	42	50.6	15.6	97.8	16.2	0.0
		(49.0–54.0)	(14.5–17.5)	(95.7–100.7)	(15.7–17.4)	(−0.4–0.7)			(48.4–53.1)	(13.9–17.7)	(94.3–101.0)	(15.7–17.4)	(−0.3–0.8)
4	144	51.8	17.3	102.4	16.4	0.2	4	104	51.2	17.1	103.1	16.3	0.1
		(50.0−54.0)	(16.0−18.7)	(100.3–106.6)	(15.6–16.9)	(−0.3–0.5)			(49.9–53.0)	(15.8–18.2)	(99.7–105.9)	(15.4–16.9)	(−0.4–0.5)
5	163	53.3	19.1	109.5	16.2	0.1	5	100	52.7	19.1	109.7	15.9	0.0
		(51.0–56.0)	(17.9–21.2)	(107.0–113.0)	(15.7–17.0)	(−0.5–0.6)			(50.6–54.5)	(17.8–20.8)	(107.0–113.0)	(15.0–16.7)	(−0.5–0.4)
6	162	55.0	22.2	116.3	16.4	0.2	6	107	53.8 **	21.3	115.8	15.8 **	−0.1 **
		(53.0–59.0)	(20.2–24.6)	(112.5–119.5)	(15.4–17.4)	(−0.3–0.7)			(51.55–57.0)	(19.4–23.9)	(112.9–119.8)	(15.1–16.7)	(−0.4–0.4)
7	150	57.0	24.8	123.0	16.5	0.0	7	185	55.5 *	23.8 *	121.5	16.0 *	−0.2 *
		(54.2–60.2)	(22.8–26.8)	(119.7–125.6)	(15.3–17.7)	(−0.4–0.6)			(53.5–58.1)	(22.1–26.3)	(118.7–125.8)	(15.1–17.2)	(−0.6–0.4)
8	162	58.4	27.4	129.2	16.4	−0.1	8	181	57.7	27.1	128.6	16.4	−0.2
		(55.7–63.0)	(24.8–31.1)	(125.8–132.2)	(15.4–18.0)	(−0.6–0.6)			(55.0–61.7)	(24.7–30.5)	(125.6–132.2)	(15.4–17.7)	(−0.6–0.4)
9	156	61.0	30.1	133.1	17.0	−0.1	9	176	60.5	30.6	133.9	16.8	−0.2
		(58.0–65.7)	(27.4–34.2)	(129.6–137.4)	(15.9–18.8)	(−0.6–0.5)			(57.0–64.1)	(27.2–34.0)	(130.1–137.7)	(15.5–18.9)	(−0.6–0.6)
10	149	63.0	33.6	139.0	17.6	−0.2	10	173	62.0	33.1	139.2	17.1	−0.3
		(59.7–69.9)	(30.4–39.4)	(134.3–142.5)	(16.1–20.0)	(−0.7–0.5)			(58.5–70.0)	(30.0–37.8)	(135.7–143.4)	(16.1–18.7)	(−0.7–0.1)
11	116	66.3	37.9	145.3	18.5	−0.2	11	183	64.9	37.0	144.7	17.7	−0.4
		(62.3–72.0)	(33.6–44.5)	(141.4–148.8)	(16.5–20.5)	(−0.7–0.5)			(60.5–71.5)	(33.6–42.4)	(140.2–148.4)	(16.1–19.9)	(−0.8–0.3)
12	83	70.2	43.9	148.2	19.5	−0.2	12	115	66.0 *	42.4	149.2	18.2 *	−0.3 *
		(65.0–77.5)	(37.4–50.8)	(144.0–153.5)	(17.6–21.8)	(−0.6–0.5)			(62.1–72.5)	(36.1–47.8)	(147.0–155.1)	(16.7–20.6)	(−0.8–0.3)

Results are presented as median (interquartile range) * *p* ≤ 0.05 vs. year 2010 (Mann–Whitney test); ** *p* ≤ 0.01 vs. year 2010 (Mann–Whitney test). Values of *n* represent the absolute number of observations in each category (i.e., volunteers of the different age analyzed in the first and the latter academic year).

**Table 3:** Anthropometric variables in girls at baseline and after 9 years.

Baseline							After 9 Years						
Age (year)	*n*	Waist Circumference (cm)	Weight (kg)	Height (cm)	BMI (kg/m^2^)	BMI Z−Score (kg/m^2^)	Age (year)	*n*	Waist Circumference (cm)	Weight (kg)	Height (cm)	BMI (kg/m^2^)	BMI Z−Score (kg/m^2^)
3	55	51.0	15.4	96.4	16.6	0.6 (−0.1–1.2)	3	33	49.0	14.9	95.7	15.9	0.1
		(48.5–54.0)	(13.8–17.1)	(93.4–99.2)	(15.7–17.4)				(47.2–53.2)	(13.2–16.4)	(93.8–99.1)	(15.1–17.0)	(−0.6–0.9)
4	147	52.5	16.8	102.1	16.2	0.4 (−0.3–0.9)	4	105	50.5 **	16.4	101.6	15.6 **	0.0 **
		(50.0–54.5)	(15.6–18.4)	(98.8–105.1)	(15.4–17.0)				(48.2–53.2)	(15.1–18.1)	(99.4–104.1)	(14.9–16.8)	(−0.5–0.7)
5	149	53.0	18.5	108.1	15.9	0.1 (−0.3–0.7)	5	117	52.5	18.6	107.9	15.8	0.1
		(51.0–55.3)	(17.2–20.4)	(104.9–111.2)	(15.2–16.9)				(50.0–55.5)	(16.8–20.6)	(104.7–112.5)	(15.0–16.8)	(−0.4–0.7)
6	167	55.0	21.4	115.4	16.0	0.1 (−0.3–0.6)	6	122	53.7 *	20.8	114.1	15.8	−0.1
		(52.2–57.9)	(19.3–23.3)	(111.7–118.3)	(15.3–16.9)				(51.5–56.6)	(18.8–22.8)	(111.6–118.1)	(15.1–16.7)	(−0.4–0.5)
7	160	57.2	24.9	122.2	16.7	0.2 (−0.4–0.9)	7	136	55.8	24.2	122.0	16.1 *	−0.1
		(53.9–62.0)	(22.3–27.6)	(118.9–125.0)	(15.2–18.2)				(53.4–59.1)	(21.8–26.9)	(118.2–126.4)	(15.2–17.4)	(−0.4–0.6)
8	165	59.0	27.4	128.0	16.7	0.1 (−0.4–0.6)	8	168	57.2	26.5	128.8	16.5	−0.2
		(56.0–64.0)	(24.8–30.9)	(124.3–131.5)	(15.8–18.2				(54.4–63.4)	(24.5–30.8)	(124.6–131.7)	(15.3–18.4	(−0.6–0.5)
9	169	61.0	30.0	132.8	17.0	−0.2 (−0.5–0.5)	9	193	59.0 *	29.5	133.5	16.8	−0.2
		(57.7–65.4)	(26.9–37.0)	(129.6–136.7)	(15.8 –18.7)				(56.1–64.2)	(26.9–34.6)	(129.4–137.3)	(15.6 –18.5)	(−0.7–0.3)
10	149	64.8	33.3	137.2	17.8	−0.1 (−0.7–0.8)	10	149	60.6 *	32.5	138.4	17.1	−0.3
		(58.1–71.0)	(29.5–39.3)	(133.1–141.6)	(15.8–20.4)				(57.1–67.0)	(29.8–38.5)	(134.5–143.1)	(15.9–19.2)	(−0.6–0.4)
11	135	64.0	37.0	145.0	17.9	−0.2 (−0.8–0.4)	11	156	61.6 *	36.4	143.8	17.4	−0.4
		(61.0–70.9)	(33.9–43.7)	(140.5–149.2)	(16.2–19.9)				(58.2–67.8)	(32.4–43.1)	(139.3–150.0)	(15.9–19.5)	(−0.9–0.2)
12	84	68.9	43.8	151.5	17.5	−0.2 (−0.6–0.5)	12	139	65.5 *	43.1	151.2	18.6	−0.2
		(64.0–74.2)	(38.4–49.2)	(146.9–156.4)	(18.8–21.1)				(60.5–70.8)	(37.2–43.1)	(146.3–156.2)	(17.2–20.6	(−0.6–0.3)

Results are presented as median (interquartile range) * *p* ≤ 0.05 vs. year 2010 (Mann–Whitney test); ** *p* ≤ 0.01 vs. year 2010 (Mann–Whitney test). Values of *n* represent the absolute number of observations in each category (i.e., volunteers of the different age analyzed during the first and the latter academic year).

**Table 4:** Global prevalence of underweight, normal weight, overweight and obesity by school type in the 2010–2019 timeframe.

Total Population		State Schools	Charter Schools	Private Schools
**International Obesity Task Force (IOTF)**	Normal weight	75.6% (5889) ^a^	80.1% (6128) ^b^	82.4% (10,088) ^c^
	Overweight	19.2% (1493) ^a^	16.4% (1253) ^b^	15.2% (1856) ^c^
	Obesity	5.3% (411) ^a^	3.6% (273) ^b^	2.4% (295) ^c^
**Faustino Orbegozo Foundation (OF)**	Underweight	0.5% (38)	0.4% (34)	0.6% (71)
	Normal weight	82.4% (6422) ^a^	86.7% (6636) ^b^	88.8% (10,870) ^c^
	Overweight	10.0% (778) ^a^	8.6% (660) ^b^	7.5% (922) ^c^
	Obesity	7.1% (555) ^a^	4.2% (324) ^b^	3.1% (376) ^c^

Data reported as percentage (*n*) per group. All differences are *p* < 0.01 (Chi-Square test). Different superscript lowercase letters indicate statistical significance in each row.

**Table 5:** Global prevalence of underweight, normal weight, overweight and obesity by school type and sex in the 2010–2019 timeframe.

			State Schools	Charter Schools	Private Schools
**Boys**	**IOTF**	Normal weight	77.3% (3134) ^a^	82.4% (3228) ^b^	83.0% (4942) ^b^
		Overweight	18.2% (739) ^a^	14.3% (560) ^b^	14.5% (865) ^b^
		Obesity	4.5% (411) ^a^	3.3% (128) ^b^	2.5% (148) ^c^
**Girls**	**IOTF**	Normal weight	73.7% (2755) ^a^	77.6% (2900) ^b^	81.9% (5146) ^c^
		Overweight	20.2% (754) ^a^	18.5% (693) ^a^	15.8% (991) ^b^
		Obesity	6.2% (230) ^a^	3.9% (145) ^b^	2.3% (147) ^c^
**Boys**	**OF**	Underweight	0.3% (14)	0.4% (14)	0.4% (24)
		Normal weight	84.8% (6422) ^a^	89.1% (3490) ^b^	89.8% (5350) ^b^
		Overweight	9.5% (385) ^a^	7.2% (281) ^b^	6.9% (412) ^b^
		Obesity	5.4% (555) ^a^	3.3% (131) ^b^	2.9% (219) ^b^
**Girls**	**OF**	Underweight	0.6% (24)	0.5% (20)	0.8% (48)
		Normal weight	79.9% (2986) ^a^	84.2% (3146) ^b^	87.8% (5520) ^c^
		Overweight	10.5% (393) ^a^	10.1% (379) ^a^	8.1% (510) ^b^
		Obesity	9.0% (336) ^a^	5.2% (193) ^b^	3.3% (206) ^c^

Data reported as percentage (*n*) per group. All differences are *p* < 0.01 (Chi-Square test). Different superscript lowercase letters indicate statistical significance in each row.
